# Characteristics of Anti-Measles Immunity in Lung Transplant Candidates

**DOI:** 10.3390/v15102121

**Published:** 2023-10-19

**Authors:** Valentina B. Polishchuk, Mikhail P. Kostinov, Aleksey A. Ryzhov, Natalia A. Karchevskaya, Irina L. Solov’eva, Alexander P. Cherdantsev, Aristitsa M. Kostinova, Arseniy A. Poddubikov

**Affiliations:** 1 Laboratory for Vaccination and Immunotherapy of Allergic Diseases, I.I. Mechnikov Research Institute of Vaccines and Sera, 105064 Moscow, Russia; 2 Department of Epidemiology and Modern Vaccination Technologies, I.M. Sechenov First Moscow State Medical University (Sechenovskiy University), 119991 Moscow, Russia; 3 Department of Thoracic and Abdominal Surgery, N.V. Sklifosovsky Research Institute for Emergency Medicine, 127994 Moscow, Russia; 4 Laboratory of Intensive Therapy and Respiratory Failure, Research Institute for Pulmonology of the Federal Medical Biological Agency, 115682 Moscow, Russia; 5 Department of Pediatrics, Ulyanovsk State University, 432017 Ulyanovsk, Russia; 6 N.V. Sklifosovskiy Institute of Clinical Medicine, I.M. Sechenov First Moscow State Medical University (Sechenovskiy University), 119991 Moscow, Russia

**Keywords:** measles, anti-measles immunity, lung transplant

## Abstract

Measles has not yet been eradicated; therefore, its outbreaks are still reported throughout the world. Like any infection, measles is dangerous for immunocompromised patients. Levels of anti-measles IgG antibodies were measured in 157 patients aged 17 to 72, who were placed on the lung transplant waiting list. Measurements were undertaken by enzyme-linked immunosorbent assay (ELISA) using the VectoMeasles-IgG kit (Russia). The proportion of patients seronegative for measles was 19% (30/157). Correlation was detected between patients’ age and their levels of anti-measles antibodies, with higher proportions of patients having undetectable titers (25.5–28.9%) or low antibody levels (38.3–44.4%) in the young age groups (17–29 and 30–39 years old). There were no differences between male and female patients in levels of anti-measles antibodies or in the proportion of seronegative individuals. Analyses of antibody levels with regard to type of disease revealed the highest rate of seronegative results in cystic fibrosis patients (34.4%, 11/32). Overall, 19% of lung transplant candidates, mostly young people and cystic fibrosis patients, did not have protective immunity against measles.

## 1. Introduction

The prevention of vaccine-controlled infectious diseases is an important part of the management plan for patients with progressive disorders affecting various organs. This is of particular importance in those cases when organ transplantation is planned due to the failure of other treatments. For recipients of solid organs, who receive life-long immunosuppressive therapy, many infections are one of the leading causes of death. Thus, this is a specific group of patients who require, both before and after organ transplantation, thorough screening for protective immunity against preventable infections [1,2,3,4,5,6].

Patients with bronchopulmonary disorders are at high risk of infection-related complications. Unlike other solid organs, the lungs are in contact with the external environment and are constantly exposed to various factors, including pathogens. In lung transplant candidates, infections lead to the exacerbation and progression of the underlying disease process, increasing the risk of death prior to transplant. Moreover, some features of innate and adaptive immunity in those patients account for the poor elimination of microorganisms from the respiratory tract [7]. The following factors predispose to post-transplant infections: impaired mucociliary clearance, denervation of the allograft, and the suppression of cough reflex [8]. In the post-transplant period, lung transplant recipients are at higher risk of infection-related complications than recipients of other organs because they receive a more aggressive immunosuppressive therapy.

Special attention should be given to infections for which live attenuated vaccines are needed for prevention. Such vaccines are not recommended after the transplantation of solid organs, meaning that patients should undergo vaccination before transplantation.

Measles is one of these infections. Despite active preventive measures, different countries have periodically faced outbreaks of this disease over the last decade. According to the World Health Organization (WHO), over 900 measles cases were reported by the end of February 2023, exceeding the number reported for all of 2022, with the Russian Federation being among the three countries that have reported the largest numbers of cases over the past 12 months. The COVID-19 pandemic has obviously contributed to an increase in measles incidence, since in many countries previously scheduled immunization campaigns were either disrupted or significantly delayed [9].

Measles virus is a potent immunosuppressive agent, and the disease is associated with a temporary but profound immunosuppression persisting over a period of several weeks to months. The wild-type measles virus infects immune cells after binding to the cellular receptor CD150, which is expressed by various populations of immune cells [10,11]. The infection and depletion of memory lymphocytes results in a loss of acquired immunological memory (immune amnesia), leading to a reduction in the levels of protective antibodies against other pathogens. M.J. Mina et al. reported that after severe or mild measles, children lost a median of 40% (range: 11 to 62%) or 33% (range: 12 to 73%), respectively, of their total preexisting pathogen-specific antibody repertoires. However, infection with the measles virus results in life-long immunity against measles itself.

These data suggest that after contracting measles infection, people could lose their immunity to previously encountered pathogens against which they have developed natural defense, as well as to those against which they have been vaccinated. Later in life, immunological memory to other infections is only restored upon a new encounter with pathogens following either natural disease or vaccination.

Unlike the wild-type measles virus, the vaccine-type virus binds to the CD46 receptor and shows a lower tropism to lymphocytes. Exposure to vaccine virus isolates induces long-term protective immunity, but is not associated with clinically significant immunosuppression [12].

Thus, patients with severe progressive disorders who are waiting for solid organ transplantation will benefit from measles vaccination because it provides protection against measles, which tends to be severe in this patient population, and prevents other vaccine-preventable infections, which are more likely to occur in patients with immunosuppression caused by measles virus. Therefore, it seems reasonable to monitor vaccine-induced immunity to vaccine-controlled infections over several years following natural disease.

Despite it being directly recommended to monitor vaccine-induced immunity and when needed to administer disease-specific booster vaccine doses to patients with comorbid conditions, in practice, this strategy is largely ignored, and such patients often lose specific immunity because of their immune deficiency. Patients with bronchopulmonary disorders are no exception. For example, among tuberculosis patients who completed a full course of measles vaccine, the proportion of individuals seronegative for measles was 36.8% [13]. Asthma has also been shown to influence the relationship between anti-measles IgG Ab levels and the time after primary vaccination. Levels of anti-measles Ab dropped over time, and the rate of this decline was higher in asthma patients compared to healthy individuals. As a result, approximately 9.3 years following primary vaccination and thereafter, the mean concentration of anti-measles antibodies and the proportion of seropositive individuals were lower in the group of asthma patients compared to those in healthy subjects [14].

A search of the literature for publications from the last decade, reporting on measles vaccination in adult patients with chronic obstructive lung disease, asthma or other bronchopulmonary disorders, did not identify any papers examining anti-measles immunity in these patient populations.

Few data could be identified in the literature regarding the development of anti-measles immunity in lung transplant candidates, depending on their disorder, age or gender. Moreover, new focal outbreaks of measles support the need to investigate this issue and to justify the necessity of vaccination for patients with various bronchopulmonary disorders.

The objective of the study was to examine the characteristics of anti-measles immunity in lung transplant candidates with severe progressive bronchopulmonary disorders.

## 2. Materials and Methods

### 2.1. Study Design and Participants

This was a cross-sectional study designed to examine the characteristics of anti-measles immunity in adult patients with severe progressive bronchopulmonary disorders, who were candidates for a lung transplant. The study was conducted at the Federal State Budgetary Scientific Institution I.I. Mechnikov Research Institute of Vaccines and Sera, the Federal State Budgetary Institution Research Institute for Pulmonology, Federal Medical and Biological Agency of Russia, and the State Budgetary Healthcare Institution N.V. Sklifosovsky Research Institute for Emergency Medicine, Moscow Department of Healthcare.

To recruit patients for the study, we used the lung transplant waiting list to select all patients who, in accordance with the requirements of the solid organ transplant program, needed an evaluation of their immunity for preventable infections and required an appropriate vaccination regimen, if they had poor or no immunity. All patients who signed an informed consent form were included in the study.

The inclusion criteria were placement on the transplant waiting list and a patient’s consent to participate in the study.

The exclusion criterion was a patient’s refusal to participate in the study.

### 2.2. Patients

The study sample included 157 patients with severe progressive bronchopulmonary disorders, aged between 17 and 72 (Me 35 (28–45)), who were placed on the lung transplant waiting list. Among them, 50.3% (*n* = 79) were males and 49.7% were females (*n* = 78). Overall, 33.1% (52/157) of the patients had obstructive pulmonary disease, 9.6% (15/157) had vascular pulmonary disease, 20.4% (32/157) had cystic fibrosis, and 36.9% (58/157) suffered from restrictive pulmonary disease. Data about previous measles vaccination were available in five subjects. The other subjects did not have documented records of having had measles or a measles vaccination.

### 2.3. Methods

Single blood samples for anti-measles IgG Ab were taken in a hospital setting. The initial evaluation of the samples was performed by a laboratory using the scientific equipment of the Collective Usage Center “I.I. Mechnikov NIIVS”. Serum levels of anti-measles IgG Ab were measured by enzyme-linked immunosorbent assay (ELISA) using the Vecto Measles-IgG kit (Vector-Best, Novosibirsk, Russia). All samples were examined in duplicate. As specified in the laboratory guidelines attached, the range of measured concentrations of the kit was 0–5 IU/mL, and the specificity and sensitivity were 100%. Serum levels of anti-measles IgG Ab equal to or exceeding 0.18 IU/mL were considered positive. Serum levels of anti-measles IgG Ab below 0.12 IU/mL were considered negative. Equivocal results (levels of IgG Ab ranging between 0.12 and 0.17 IU/mL) were considered negative. For serum levels of IgG Ab, the following three groups were arbitrarily identified: low (0.18–1.0 IU/mL), moderate (1.0–5.0 IU/mL) and high (above 5.0 IU/mL).

### 2.4. Statistical Analysis

Descriptive statistics for quantitative variables included the median and interquartile range, while descriptive statistics for qualitative variables included proportions and their 95% confidence intervals estimated using the Clopper–Pearson method and numbers of subjects with the specified characteristics. The intergroup comparison of qualitative variables was performed using the χ^2^ test. For cells with an expected frequency of less than 5%, analysis was done using the Fisher’s exact test. The comparison of quantitative variables was carried out using the Mann–Whitney test for two independent samples and the Kruskal–Wallis test for three independent samples. Post-hoc comparisons were conducted applying the Dunn procedure. All calculations were undertaken using the open source statistical programming environment R (Project R for statistical computing) (v. 4.0.4).

## 3. Results

**Figure 1:**
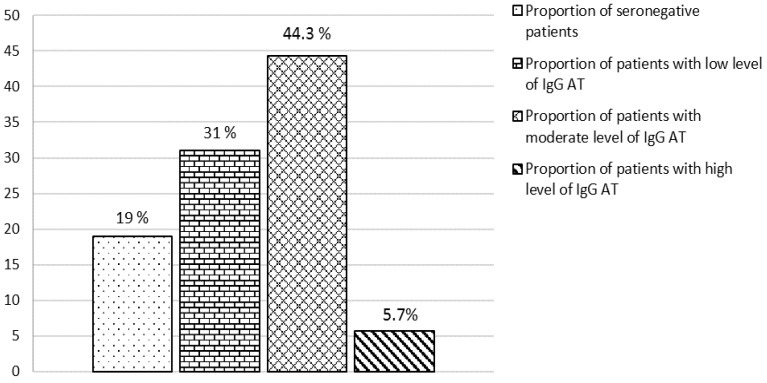
Disposition of patients with severe progressive bronchopulmonary disorders by level of anti-measles IgG antibodies.

The analysis of serum samples taken from 157 lung transplant candidates who were placed on the transplant waiting list showed that 19% (30/157) of the patients were seronegative for measles. Of these, 90% (27/30) had negative results (anti-measles IgG Ab below 0.12 IU/mL) and 10% (3/30) had equivocal results (anti-measles IgG Ab 0.12–0.18 IU/mL) (Figure 1). The median level of anti-measles IgG Ab was 0.95 (0.26-1.96) IU/mL. Most patients (44.3%) showed moderate levels of anti-measles IgG Ab, ranging between 1.0 and 5.0 IU/mL.

### 3.1. Analysis of the Relationship between Levels of Anti-Measles IgG Antibodies and Age in Patients with Severe Progressive Bronchopulmonary Disorders

For more detailed analysis, the patients were divided into the following four age groups: 17–29, 30–39, 40–49 and 50 years and older. An analysis of the relationship between levels of anti-measles IgG Ab and age showed that patients from the younger age groups (17–29 and 30–39) had significantly lowers antibody levels than those from the older age groups (40–49 and >50) (Table 1).

**Figure 2:**
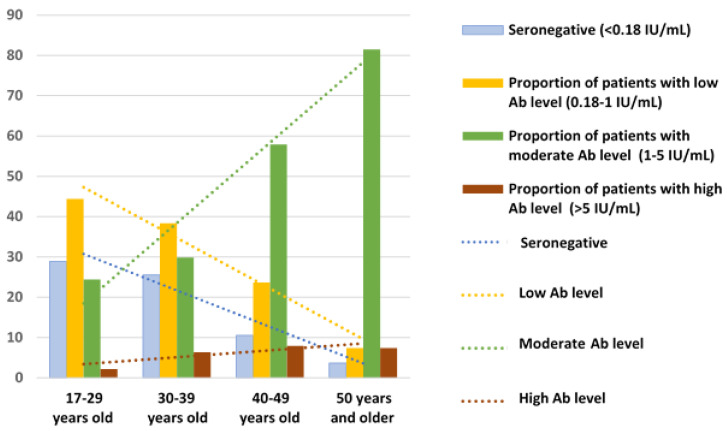
Distribution by age of anti-measles IgG Ab levels in patients with severe progressive bronchopulmonary disorders.

Our analysis also revealed lower proportions of patients with seronegative results and low antibody levels, and respectively higher proportions of those with moderate and high levels of IgG Ab (>5.0 IU/mL) in older age groups (Table 1 and Figure 2).

**Figure 3:**
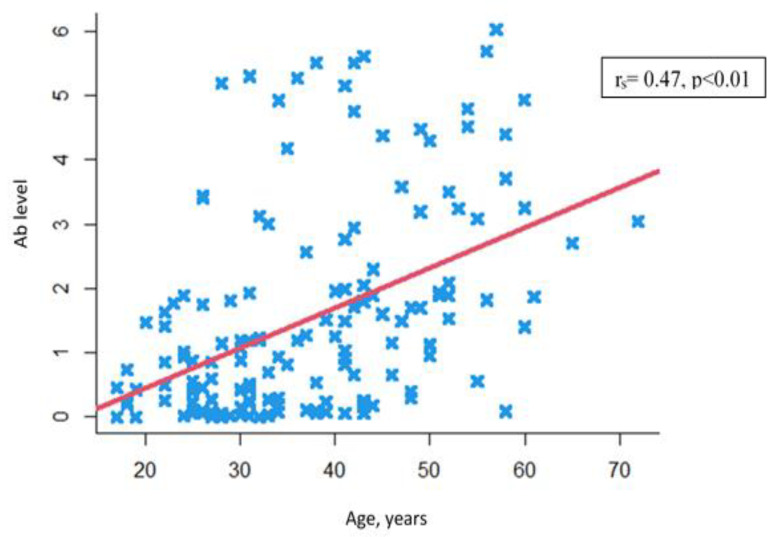
Correlation between anti-measles IgG Ab levels and patients’ age. 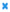
—Individual values.

Another finding was a positive statistically significant correlation between levels of anti-measles antibodies and patients’ age (r_s_ = 0.47, *p* < 0.01) (Figure 3).

### 3.2. Analysis of the Relationship between Levels of Anti-Measles IgG Antibodies and Gender in Patients with Severe Progressive Bronchopulmonary Disorders

**Figure 4:**
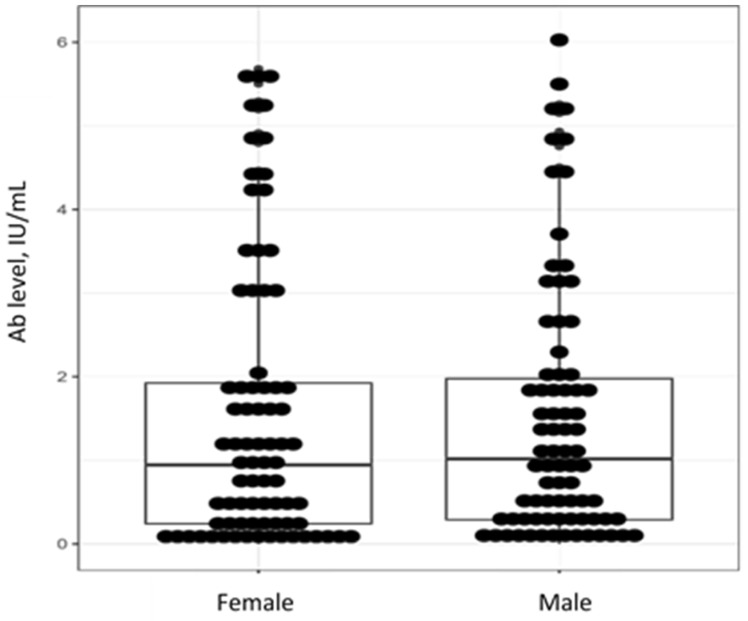
Individual values, medians and interquartile ranges for levels of anti-measles IgG Ab by gender in patients with severe progressive bronchopulmonary disorders.

The male and female study subjects were matched by age (39 (27–48) years old vs. 33 (28–43) years old (*p* = 0.204)). Non-age-adjusted levels of anti-measles IgG Ab did not differ between men and women (W = 3162.5, *p* = 0.7761) (Figure 4).

Similarly, no gender differences were detected in the subgroups identified by antibody level (Table 2). Most males and females (45.6% and 42.3%, respectively) had moderate levels of anti-measles IgG Ab (1.0–5.0 IU/mL).

### 3.3. Age- and Gender-Adjusted Analysis of Anti-Measles IgG Antibodies in Patients with Severe Progressive Bronchopulmonary Disorders

Age- and gender-adjusted analyses revealed that both males and females from the 17–29 and 30–39 age groups had significantly lower levels of anti-measles IgG Ab than gender-matched patients in the oldest age group (50 years and older) (Table 3).

Among the 30 seronegative study subjects, 43% (13/30) were males and 57% (17/30) were females (χ^2^ = 0.658 *p* = 0.418); 7 patients (23%) had obstructive pulmonary disease, 2 patients (7%) had vascular pulmonary disease, 11 patients (37%) had cystic fibrosis, and 10 patients (33%) suffered from restrictive pulmonary disease.

Among seronegative patients, there were more women than men in the 17–29 age group (*p* = 0.047), while in the other age groups there were no such gender differences (Table 4).

Another finding was a statistically significant positive correlation between levels of anti-measles antibodies and patients’ gender. The correlation coefficient was r_s_ = 0.54, *p* < 0.01 in the female subgroup and r_s_ = 0.41, *p* < 0.01 in the male subgroup.

### 3.4. Analysis of Anti-Measles IgG Antibodies by Type of Pulmonary Disease

The distribution of patients by type of disease shows that patients with vascular lung disease and cystic fibrosis were significantly younger than those with restrictive or obstructive lung disease. As expected, younger patients with restrictive or obstructive lung disease had considerably lower levels of anti-measles IgG Ab than older patients (Table 5).

The highest proportion of patients seronegative for measles were in the cystic fibrosis group (34.4%; 11/32). In the groups of patients with obstructive, vascular and restrictive pulmonary disease, these proportions were 13.5% (7/52), 13.3% (2/15) and 17.2% (10/58), respectively. Significant differences were revealed between the groups of patients with obstructive pulmonary disease and cystic fibrosis (χ^2^ = 3.9788, *p* = 0.046). Further analyses of anti-measles antibody levels with regard to type of pulmonary disease revealed no other differences.

## 4. Discussion

In spite of mass primary measles vaccination and subsequent booster vaccination in children, both immunocompromised and immunocompetent adult patients have no evidence of long-term protective vaccine-induced immunity [15,16,17,18,19,20,21]. In a meta-analysis conducted by A.V. Nozdracheva et al., the proportions of individuals with seronegative anti-measles antibody levels among healthy adults aged between 18 and 60 ranges between 23.4% and 29%, with a predominance of seronegative individuals (40.4–45.3%) among young adults [22], while one of the provisions for a favorable epidemiological situation is no more than 7% seronegative results among the vaccinated population [23]. In a study, only 32% of healthy adults, aged between 18 and 30, had protective levels of anti-measles antibodies [24]. According to foreign authors, herd immunity rates ranges between 84.6% and 89.9%, also with a predominance of seronegative individuals in young adults [25,26,27]. Even among healthcare providers, the proportion of individuals with seronegative or equivocal anti-measles antibody levels ranges between 14.6% and 37%, reaching 38.5–70% in some age groups [18,19,28]. In a study conducted by T.G. Tkachenko et al., 63% of the healthcare institution staff was seropositive for measles, while in the younger age groups (20–29 and 30–39), this proportion reached 61–70% [18]. Similar data were reported in studies conducted in other countries, where the proportion of seronegative subjects amongst medical providers ranged between 13% and 46%, with this percentage being the highest among young people [29,30,31,32].

In our study of anti-measles immunity in patients with severe progressive bronchopulmonary disorders, the proportion of seronegative patients was 19%. This is comparable to the proportion of seronegative individuals in a healthy population [16,19,22]. According to foreign authors, the proportion of seronegative individuals among candidates for organ transplantation ranges between 1.4% and 13.2% [33,34,35,36].

Age-adjusted analysis in the subset of seronegative lung transplant candidates showed that in the 17–39 age group, the proportion of seronegative patients was 25.5–28.9%, which was significantly higher than in the group of patients aged 50 years and older (3.7%). Similar results were obtained in studies evaluating levels of IgG Ab in healthy people, including healthcare workers of maternity hospitals, aged 21–43 (22.7%), and parturient women (21.4%) [19,28]. An age-adjusted analysis of anti-measles immunity in the staff of a large healthcare institution showed that the proportions of seronegative and equivocal results ranged between 0% in the oldest age group (64 years and older) and 38.5% in the youngest age group (19–23 years old) [28]. Our study in patients with severe progressive bronchopulmonary disorders yielded similar results, showing that older age groups had lower proportions of patients with seronegative results and low anti-measles antibody levels, and respectively higher proportions of patients with moderate IgG Ab levels. It could be assumed that high percentages of seronegative subjects in young age groups are associated with mass primary and booster measles vaccination campaigns, which led to a reduction in the number of infection spots and outbreaks of this disease. In 95–100% of cases, primary and booster measles vaccinations given at an early age fail to provide long-term protection in the context of reduced circulation of the measles virus and the lack of immune enhancement by asymptomatic infection. Because of limited natural boosting, vaccine-induced immunity is maintained over shorter periods of time, which explains the higher percentages of seronegative patients among people under 40–45 years of age, as reported in our study. According to the results of a recently published study, the average rate of waning immunity against measles was 9.7% per year after the first dose of MMR vaccine and 4.8% after the second dose [37]. Older people (aged 46 and older) have high titers of IgG Ab, regardless of their health status. This is explained by infection-induced immunity, received by the population before the stage of mass immunization, which is maintained almost throughout life. In a study conducted by N. Friedrich et al., more than 97% of adults born before 1965, but only 74–76% of adults born between 1975 and 1993, were seropositive for anti-measles IgG [26]. Data reported by F.P. Bianchi et al. provide further evidence that natural immunity is more long-lasting than vaccine immunity. The authors reported that among young people, aged 20 to 25, the proportion of seronegative subjects was 20% in those who received two doses of measles vaccine in childhood and only 6% in those with a history of measles [38]. The level of protection after primary vaccination and revaccination in people with compromised health may be reduced, that is, they lose the ability to maintain protective immunity over time. Schulman et al. reported an immune response to measles vaccine in 80% of children on dialysis and in 98.3% of healthy children [39]. In a paper published in 1996, the authors also demonstrated that people with health problems (such as allergy, diabetes mellitus, metabolic disorders) developed antibodies more slowly and lost their specific immunity faster than healthy subjects [40].

In a study of anti-measles immunity conducted by H. Hostetler et al. in a group of lung transplant candidates, gender-adjusted analysis showed that there were higher odds of undetectable titers associated with the female sex [34]. In our study, gender-adjusted analysis did not reveal any evident differences between males and females in antibody levels or the proportion of seronegative individuals. However, after having adjusted for both gender and age, the analysis of antibody levels revealed that in the youngest age group (17–29 years old), the proportion of seronegative females was higher than that of males: 43.5% (21.5–69.2) females vs. 13% (2.91–34.9) males (*p* = 0.047).

After adjustment for type of disease, the analysis of antibody levels revealed that among our patients with severe progressive bronchopulmonary disorders, the highest proportion of seronegative results was in the subjects with cystic fibrosis (34.4%). H. Hostetler et al. reported similar results, showing that undetectable titers of anti-measles antibodies were more often reported in cystic fibrosis compared to non-cystic fibrosis patients (19.1% vs. 9.6%). The authors suggested that cystic fibrosis may result in specific immune defects due to a defective cystic fibrosis transmembrane conductance regulator (CFTR) on the cell membrane, leading to increased titer loss over time and impaired vaccine response [34]. Of note, immunity induced by bacterial vaccines is also underdeveloped in these patients, who show poor clinical responses to vaccination, i.e., no reduction in the rates of respiratory infections or cystic fibrosis exacerbations, or in the number of antibacterial courses given in addition to background treatment [41]. This might suggest that cystic fibrosis patients require a more frequent evaluation of their anti-measles IgG Ab levels, and should receive booster vaccination when needed. Anti-measles vaccines can also be given during treatment with immune-active agents, which are able to booster vaccine-induced immunity in individuals with cellular and/or humoral immune abnormalities [42].

It should be emphasized that vaccination during treatment with immune-active agents not only induces an antibody response comparable to that in healthy people, but also helps maintain this response over a longer period. In other studies, patients who frequently suffered from respiratory tract infections, leading to exacerbations of chronic illnesses, received either bacterial lysate (I.R.S. 19) or recombinant interferon alpha-2b (Viferon^®^) as medication support for 14 days after booster vaccination against measles and mumps. This helped to prevent acute respiratory infections in these patients and contributed to a good vaccine-induced immune response. Six months after booster vaccination, there were no individuals with undetectable anti-measles antibody titers in these patient populations, and 87.5–100% of the patients had moderate or high protective IgG Ab levels (which were not different from those in healthy people). In the comparison group, which included patients who received booster vaccine but did not receive medication support, the proportion of individuals with undetectable anti-measles antibody titers was 26.9% [43]. Other authors also reported data suggesting that immune-active agents of various types might improve the production of specific anti-measles antibodies [44,45,46,47].

## 5. Conclusions

Our study showed that 19% of lung transplant candidates with severe progressive bronchopulmonary disorders, mostly young people and cystic fibrosis patients, did not have protective levels of anti-measles IgG Ab, suggesting the need for booster vaccination.

## Figures and Tables

**Table 1:** Levels of anti-measles IgG Ab, Me (Q1–Q3) and their distribution by age in patients with severe progressive bronchopulmonary disorders.

Age Group	Anti-Measles IgG Ab, Me (Q1–Q3)	Distribution of Patients by Level of IgG Ab *			
		Seronegative	Low Ab Level	Moderate Ab Level	High Ab Level
Group 1 17–29 (*n* = 45)	0.43 (0.094; 1.02)	28.9 (16.4–44.4)	44.4 (29.6–60)	24.4 (12.9–39.5)	2.2 (0.06–11.8)
Group 2 30–39 (*n* = 47)	0.48 (0.15; 1.22)	25.5 (13.9–40.4)	38.3 (24.5–53.6)	29.8 (17.3–44.9)	6.4 (1.3–17.5)
Group 3 40–49 (*n* = 38)	1.65 (0.65; 2.76)	10.5 (2.9–24.8)	23.7 (11.4–40.2)	57.9 (40.8–73.7)	7.9 (1.7–21.4)
Group 4 50 years and older (*n* = 27)	2.71 (1.52; 4.3)	3.7 (0.09–19)	7.4 (0.9–24.3)	81.5 (61.9–93.7)	7.4 (0.9–24.3)
Comparison between age groups	H = 37.18, *p* < 0.001	χ^2^ (3, N = 157) = 34.78, *p* < 0.0001			
	*p*^1–2^ = 0.99 *p*^1–3^ < 0.001 *p*^1–4^ < 0.001 *p*^2–3^ = 0.017 *p*^2–4^ < 0.001 *p*^3–4^ = 0.119	*p*^1–4^ = 0.021 *p*^2–4^ = 0.024	*p*^1–3^ = 0.048 *p*^1–4^ < 0.001 *p*^2–4^ = 0.009	*p*^1–3^ = 0.002 *p*^1–4^ < 0.001 *p*^2–3^ = 0.009 *p*^2–4^ < 0.001	

* The proportions of subjects with the specified characteristic and their 95% confidence intervals estimated using the Clopper–Pearson method. *p*^1–2^ was the value for comparison of Ab levels between age groups 1 and 2, p^1–3^ was the value for comparison of Ab levels between age groups 1 and 3, p^1–4^ was the value for comparison of Ab levels between age groups 1 and 4, p^2–3^ was the value for comparison of Ab levels between age groups 2 and 3, p^2–4^ was the value for comparison of Ab levels between age groups 2 and 4, p^3–4^ was the value for comparison of Ab levels between age groups 3 and 4.

**Table 2:** Levels of anti-measles IgG Ab, Me (Q1–Q3), and their distribution by gender (%), in patients with severe progressive bronchopulmonary disorders.

Patient Group	Anti-Measles IgG Ab, Me (Q1–Q3)	Distribution of Patients by Level of IgG Ab *			
		Seronegative	Low Ab Level	Moderate Ab Level	High Ab Level
Male (*n* = 79)	1.02 (0.28; 1.99)	16.5 (9.1–26.5)	32.9 (22.7–44.4)	45.6 (34.3–57.2)	5.1 (1.4–12.5)
Female (*n* = 78)	0.94 (0.25; 1.94)	21.8 (13.2–32.6)	29.5 (19.7–40.9)	42.3 (31.2–54)	6.4 (2.1–14.3)
Comparison	*p* = 0.78	χ^2^ (1, N = 157) = 0.95, *p* = 0.81			

* The proportions of subjects with the specified characteristic and their 95% confidence intervals estimated using the Clopper–Pearson method.

**Table 3:** Levels of anti-measles IgG Ab, Me (Q1-Q3) by age and gender in patients with severe progressive bronchopulmonary disorders.

Age Group		Gender		*p* Level
		Male	Female	
Group 1 17–29 years old (*n* = 45)	IgG Ab level	0.67 (0.24–1.41)	0.26 (0.06–0.85)	*p* = 0.11
	n	22	23	
Group 2 30–39 years old (*n* = 47)	IgG Ab level	0.36 (0.08–1.1)	0.69 (0.25–1.22)	*p* = 0.27
	n	18	29	
Group 3 40–49 years old (*n* = 38)	IgG Ab level	1.49 (0.3–2.3)	1.7 (1.03–2.95)	*p* = 0.36
	n	21	17	
Group 4 50 years and older (*n* = 27)	IgG Ab level	2.4 (1.4–3.71)	3.09 (1.88–4.3)	*p* = 0.5
	n	18	9	
Comparison		*p*^1–4^ < 0.01 *p*^2–4^ < 0.001	*p*^1–3^ < 0.01 *p*^1–4^ < 0.001 *p*^2–4^ < 0.01	

*p*^1–3^ was the value for comparison of Ab levels between age groups 1 and 3, *p*^1–4^ was the value for comparison of Ab levels between age groups 1 and 4, *p*^2–4^ was the value for comparison of Ab levels between age groups 2 and 4.

**Table 4:** Gender- and age-adjusted proportions of individuals seronegative for measles in patients with severe progressive bronchopulmonary disorders.

Age Group		Proportion of Seronegative Patients		*p* Level *
		Male	Female	
Group 1 17–29 years old (*n* = 45)	%	13 (2.91–34.9)	43.5 (21.5–69.2)	*p* = 0.047
	*n*/N	3/22	10/23	
Group 2 30–39 years old (*n* = 47)	%	33.3 (13.3–65.8)	20.7 (8–39.7)	*p* = 0.49
	*n*/N	6/18	6/29	
Group 3 40–49 years old (*n* = 38)	%	14.3 (3–36.3)	5.9 (0.1–28.7)	*p* = 0.61
	*n*/N	3/21	1/17	
Group 4 50 years and older (*n* = 27)	%	5.6 (0.1–27.3)	0 (0–33.6)	*p* = 1.0
	*n*/N	1/18	0/9	
Comparison		χ^2^ = 5.4844 *p* = 0.14	*p*^1–3^ < 0.011 *p* ^1–4^ = 0.03	

* Comparisons were performed using the two-tailed Fisher’s exact test. *p*^1–3^ was the value for comparison of Ab levels between age groups 1 and 3, *p*^1–4^ was the value for comparison of Ab levels between age groups 1 and 4.

**Table 5:** Levels of anti-measles IgGAb, Me (Q1–Q3) and their distribution (%) by type of disease in patients with severe progressive bronchopulmonary disorders.

Type of Disease	Age	Anti-Measles IgG Ab, Me (Q1-Q3)	Distribution of Patients by Level of IgG Ab *			
			Seronegative	Low Ab Level	Moderate Ab Level	High Ab Level
1. Obstructive (*n* = 52)	41 (32.5–47.5)	1.22 (0.27–1.97)	13.5 (5.6–25.8)	32.7 (20.3–47.1)	48.1 (34–62.5)	5.8 (1.2–15.9)
2. Vascular (*n* = 15)	30 (20–35)	0.66 (0.43–1.75)	13.3 (1.7–40.5)	40 (16.3–67.7)	46.7 (21.3–73.4)	0 (0–21.8)
3. Cystic fibrosis (*n* = 32)	26 (24–28)	0.34 (0.08–0.97)	34.4 (18.6–53.2)	40.6 (23.7–59.4)	21.9 (9.3–40)	3.1 (0.08–16.2)
4. Restrictive (*n* = 58)	41 (32–50)	0.98 (0.26–1.96)	17.2 (8.6–29.4)	22.4 (12.5–35.2)	51.7 (38.2–65)	8.6 (2.9–19)
Comparison	*p*^1–2^ < 0.049 *p*^1–3^ < 0.001 *p*^2–4^ < 0.0085 *p*^3–4^ < 0.001	H = 12, *p* = 0.01 *p*^1–3^ < 0.024 *p*^3–4^ < 0.002	*p* = 0.096	*p* = 0.26	*p* = 0.043	*p* > 0.05

* The proportions of subjects with the specified characteristics and their 95% confidence intervals were estimated using the Clopper–Pearson method. *p*^1–2^ was the value for comparison of Ab levels in patients with obstructive pulmonary diseases and patients with vascular diseases, *p*^1–3^ was the value for comparison of Ab levels in patients with obstructive pulmonary diseases and patients with cystic fibrosis, *p*^2–4^ was the value for comparison of Ab levels in patients with vascular diseases and patients with restrictive pulmonary diseases, *p*^3–4^ was the value for comparison of Ab levels in patients with cystic fibrosis and patients with restrictive pulmonary diseases.

## Data Availability

Data available upon request from the authors.
